# Stress and Quality of Life of Patients with Cancer: The Mediating Role of Mindfulness

**DOI:** 10.1155/2020/3289521

**Published:** 2020-12-10

**Authors:** Mahlagha Dehghan, Moazame Jazinizade, Alireza Malakoutikhah, Ali Madadimahani, Mohammad Hossein Iranmanesh, Shahriar Oghabian, Fatemeh Mohammadshahi, Fatemehzahra Janfaza, Mohammad Ali Zakeri

**Affiliations:** ^1^Nursing Research Center, Kerman University of Medical Sciences, Kerman, Iran; ^2^Student Research Committee, School of Nursing and Midwifery, Kerman University of Medical Sciences, Kerman, Iran; ^3^Student Research Committee, School of Medicine, Kerman University of Medical Sciences, Kerman, Iran; ^4^Student Research Committee, School of Dentistry, Kerman University of Medical Sciences, Kerman, Iran; ^5^Social Determinants of Health Research Centre, Non-Communicable Diseases Research Center, Rafsanjan University of Medical Sciences, Rafsanjan, Iran

## Abstract

**Background:**

Cancer is one of the major health problems worldwide, which in addition to physical disorders, causes stress and anxiety in patients and affects the quality of life of cancer patients. Mindfulness can affect stress and improve the quality of life. This research explained the correlation between stress, quality of life, and mindfulness.

**Materials and Methods:**

Two hundred five cancer patients participated in this cross-sectional study. Patients completed the EORTC Quality of Life Questionnaire Core 30 (EORTC QLQ-C30), the Mindfulness Attention and Awareness Scale (MAAS), and Perceived Stress Scale (PSS).

**Results:**

Perceived stress and mindfulness predict nearly 39% of the changes of QOL in cancer patients. In addition, perceived stress was negatively associated with mindfulness and quality of life (*P* < 0.05). Mindfulness was positively correlated with quality of life (*P* < 0.05). Mindfulness played a mediating role in the relationship between perceived stress and quality of life (standardized *β* = −0.13; SE = 0.07, 95% confidence interval = −0.28 to −0.01; *P* value = 0.04).

**Conclusion:**

In the present study, the variables of mindfulness and perceived stress affected the quality of life of cancer patients. Mindfulness can affect the quality of life of cancer patients directly and indirectly. These results emphasize the importance of mindfulness in the lives of cancer patients.

## 1. Introduction

Cancer is a major health problem worldwide, and the US has the second highest rate of victims [1]. The World Health Organization declared 10 million cancer patients in 2000, which is expected to go up to 15 million by 2020, with 60% of them in underdeveloped countries [2].

Cancer and its treatments often cause physical and mental disorders in patients, and the biggest problem of the cancer patients is the stress and anxiety caused by the disease. According to statistics, one in three people with cancer suffer from significant psychological disorders, which reduce the quality of treatment and their recovery [3–5]. One of the problems of the cancer patients is the stress of coping with the disease. Diagnosing, treating, and living with cancer can all be very stressful. Stress can affect the development, progression, and metastasis of cancerous tumors. If the stress experienced by the patient is not managed properly, it will have a great impact on his/her health [3, 4, 6]. The results of clinical studies support the correlation between stressful events and the survival and improvement of cancer. Despite insufficient knowledge about specific mechanisms involved in stress-induced tumorigenic processes, clinical studies have shown that stress can lead to cancer progression [7].

Stress is often defined as an internal or external challenge, disorder, or stimulus; others perceive stress as a physiological challenge or response [3]. Stress is the correlation between a person and a health-threatening environment [4]. Chronic stress causes changes in the hormone levels and has negative physiological effects on the body [7]. Stress has adverse effects on the health, and it is one of the most important factors in the progression and development of the cancer. Growing evidence suggests that stress can affect the neuroendocrine system and exacerbate tumor activity [8]. Short-term stress lasts from a few minutes to several hours (for example, job interviews, prereading before lectures, and sports activities), and chronic stress is defined as long-term stresses (such as patient care, communication problems, and long-term financial problems) [3].

Stress affects many aspects of the cancer patients' life, including their quality of life. Quality of life includes all aspects of life experiences, illnesses, and treatment. The cancer patients' quality of life changes over time, and the poor quality of life of the chronic patients is one of the consequences of stress [3, 4, 6]. Kwon et al. showed that the quality of life of patients was significantly lower three years after diagnosis compared with the time of diagnosis [9]. DeNysschen et al. also showed that improving the quality of life was one of the most important measures to increase survival of those recovered from breast cancer [10].

Today, patients are interested in using other methods such as meditation and psychological methods along with pharmaceutical methods [11]. Mindfulness is one of the methods affecting stress [12]. Mindfulness is the quality of being present and fully engaged with whatever we are doing now without judgment. Mindfulness varies from one moment to another and from one person to another [13]. A mindful person pays attention to the present and does not consider the past and the future [14]. Therefore, a mindful person does not lose his/her direct connection with reality and intrapsychic and environmental events [13].

Mindfulness can reduce stress and anxiety; improve sleep disorders; reduce depression and stress, self-harm, and aggressive behaviors, increase patience and relaxation in the treatment process; create enthusiasm and motivation to fight against disease; improve quality of life; reduce pain and suffering caused by illness; and strengthen the immune system [11, 12, 15–23]. In fact, meditation increases the feeling of well-being [24]. In addition, mindfulness-based interventions reduce psychological symptoms and have positive effects on health outcomes such as experiencing positive emotions, gaining effective coping skills, purposeful thinking in life, and reducing emotional exhaustion [25, 26]. Tate et al. systematically studied qualitative evidence of cancer patients' attitudes toward mindfulness and found that the evolution of mindfulness practice created a new and alternative perspective on how patients were able to change their perceptions of themselves and the world around them [27].

Mindfulness plays a special role in explaining the important components of mental health of cancer patients. Pour and Kord have shown that mindfulness was correlated with the quality of life of the cancer patients. Mindful people evaluate life-threatening situations with less stress and are more adaptable to stressful situations [28]. Zhong et al. showed that the higher the mindfulness, the lower the psychological symptoms of the cancer patients [29]. Studies have shown that mindfulness can also improve positive emotions, sense of energy, and happiness in depressed people, which can play a role in increasing the quality of life of these people. Although some studies have shown that mindfulness improves the quality of life of cancer patients [6, 30, 31], Lengacher et al. indicated that mindfulness improved stress and anxiety of the cancer patients rather than their quality of life; and further research would be needed [32].

Many researchers have considered mindfulness and its positive effects in various fields, including cancer treatment. However, according to the review of literature, limited studies have examined the correlation between stress, quality of life, and mindfulness and the mediating role of mindfulness in the relationship between stress and quality of life. Therefore, this study aimed to investigate the correlation between stress, quality of life, and mindfulness of the cancer patients in southeastern Iran.

## 2. Methods

### 2.1. Study Design and Setting

This was a cross-sectional and descriptive analytical study. The research setting was the oncology ward of Bahonar Hospital and Javadalaimeh Clinic in Kerman. These are the main centers for providing services to patients with cancer in the city of Kerman and southeastern Iran.

### 2.2. Sample Size and Sampling

The study population consisted of male and female patients with cancer referred to the research setting. Patients adhering to the inclusion criteria were considered eligible to participate in the study.

Inclusion criteria: (a) patients aged over 18 years (to have a correct understanding of the questions); (b) patients diagnosed with cancer; and (c) awake and aware patients.

Exclusion criteria: (a) patients psychologically and physiologically unstable during sampling and (b) patients not able to complete more than 10% of each of the questionnaires.

The following formula and the study of Zhong et al. were used to estimate the sample size [29]:

(1) $$\begin{array}{c} n={\left(\frac{z_{1-\left(\alpha /2\right)}+z_{1-\beta }}{0.5\;\ln\left(\left(1+r\right)|/|\left(1-r\right)\right)}\right)}^{2}+3. \end{array}$$

The type I and type II errors were considered 0.05% and 20%, respectively. According to the results of Zhong et al., the correlation coefficient between mindfulness and general health of the patients with cancer was considered 0.20. According to the aforementioned indicators, 205 samples were included in this study. Concerning the probability of dropouts, 255 questionnaires were provided to the eligible samples, of which 240 questionnaires were completed. Therefore, the response rate was 94.11%. Furthermore, out of 240 completed questionnaires, 35 questionnaires were removed because of missing values. Finally, 205 questionnaires were statistically analyzed. Convenience sampling method was used.

### 2.3. Measures

#### 2.3.1. Demographic Characteristics Form

This form included age, gender, marital status, educational level, occupation, monthly income, cancer type, cancer stage, duration of diagnosis, type of treatment, and other diseases.

#### 2.3.2. Mindfulness Attention and Awareness Scale

The Mindfulness Attention and Awareness Scale (MAAS) was developed by Ryan and Brown in 2003 to measure mindfulness. This scale consists of 15 questions with a 6-point Likert scale (1 = almost always, 6 = almost never). The minimum score of this scale is 15, and the maximum score is 90. The higher the score, the higher the level of mindfulness [33].

The MAAS shows a good internal consistency. Its Cronbach's alpha was reported to be 0.82 for the student sample and 0.87 for the adult sample. The MAAS has a convergent validity with Mindfulness/Mindfulness Scale (MMS) and Rosenberg Self-Esteem Scale (RSS). In addition, the MAAS has a divergent validity with Beck Depression Inventory (BDI) and the State-Trait Anxiety Inventory (STAI) [33]. In the study of Ghorbani et al., the Cronbach's alpha of its Persian version was 0.81 in 723 student samples. In terms of validity, the Persian version of this scale had a significant correlation with the Internal State Awareness (ISA) [34].

#### 2.3.3. Perceived Stress Scale (PSS)

Cohen et al. developed the Perceived Stress Scale (PSS) in 1983 to measure the perception of stress, thoughts, and feelings about stressful events, controlling, overcoming, and coping with stress over the past month [35]. The scale is self-administered and has 14 items. Each item is scored by a five-point Likert scale (0 = never, 1 = almost never, 2 = sometimes, 3 = often, 4 = always). Items 4, 5, 6, 7, 9, 10, and 13 are scored reversely. The total score of the scale is obtained from the sum of the scores of all the items. The lowest score on this scale is 0, and the highest is 56. The higher the score, the more perceived the stress [35]. A study has mentioned that this scale has two subscales of positively worded and negatively worded [36].

According to Cohen et al., the Cronbach's alpha coefficient for reliability of this scale was 0.84. The PSS has a convergent validity with the Life-Event Scale. For its criterion validity, the correlation coefficient of the scale was between 0.52 and 0.76 using the Symptomatological Measures [35]. In the study of Behrozy et al., the Cronbach alpha and split-half coefficient were 0.73 and 0.74, respectively. The construct validity coefficient of this scale was 0.63 according to a researcher-made criterion, and the significance level was *p* < 0.05 [37].

#### 2.3.4. Quality of Life Questionnaire-Core 30 (QLQ-C30)

Quality of Life Questionnaire-Core 30 (QLQ-C30) was developed by the European Organization for Research and Treatment of Cancer (EORTC) in 1987 to measure the quality of life of the patients with cancer [38]. The QLQ-C30 has four versions (QLQ-C30 version 1.0, QLQ-C30 (+3) interim version, QLQ-C30 version 2.0, and QLQ-C30 version 3.0). The QLQ-C30 version 3.0 is currently the standard version [39].

Version 3.0 of the questionnaire was designed to measure cancer patients' physical, psychological, and social functions. The questionnaire is composed of 5 multi-item scales (physical, role, social, emotional, and cognitive functioning) and 9 single items (fatigue, pain, nausea/vomiting, airway obstruction, insomnia, loss of appetite, constipation, diarrhea, and financial problems). Items 1 to 28 were scored based on a 4-point Likert scale (1 = never, 4 = too much), and items 29 and 30 were based on a 7-point Likert scale (1 = very poor, 7 = excellent). The scores range from 0 to 100. In the dimensions of function and overall quality of life, the higher the score, the better the function or quality of life. In the dimension of symptoms, the higher the score, the greater the symptoms [39].

Aaronson et al. studied QLQ-C30 version 3.0 on patients with narcotic cancer. The reliability of all dimensions was good (Alpha Cronbach > 0.7). In addition, the questionnaire had a proper validity [38]. Safaee et al. measured the validity and reliability of the Persian version of QLQ-C30 v. 3.0 in patients with breast cancer. The questionnaire had a good convergent validity in all dimensions (*r* > 0.4). Divergent validity has been reported in all items except for physical functioning (item 4). The questionnaire had a good reliability in most dimensions (Cronbach alpha >0.7), except for fatigue (0.65), pain (0.69), and nausea/vomiting (0.66), which reliability was less than 0.7. However, they have been in an acceptable range [40].

### 2.4. Data Collection Procedure

After receiving the code of ethics and obtaining permissions from “REDACTED,” researchers referred to the research setting. They explained the goals and methodology of the study to the eligible patients. For those patients who were unaware of their disease, a researcher asked the questions orally and filled the questionnaire. In case one sample had not been able to cooperate the sampling, his/her companion would have helped him/her. Sampling lasted from January to February 2020.

### 2.5. Statistical Analysis

SPSS 20 and AMOS 24 were used for data analysis. Descriptive statistic (frequency, percentage, mean, and standard deviation) was used to describe the characteristics of the participants. Independent *t*-test, Mann–Whitney U, Kruskal–Wallis H, and analysis of variance (ANOVA) were used to examine the mean differences in mindfulness according to the qualitative variables. The Pearson correlation coefficient was used to investigate the correlation between mindfulness, perceived stress, and quality of life. Furthermore, the structural equation modeling method was used to test the proposed relationship between mindfulness, perceived stress, and quality of life. The Mahalanobis d^2^ index was examined to check the multivariate outliers. Accordingly, six outliers were excluded from the analysis. The univariate normality of the main variables was checked using the skewness and kurtosis indices. The multivariate normality was checked using Mardia's normalized multivariate kurtosis value, which was 1.39. Therefore, the multivariate normality was confirmed [41–43]. Model adequacy was evaluated by the chi-square test. The main model fit indices were the Goodness of Fit Index (GFI), Incremental Fit Index (IFI), Parsimonious Comparative Fit Index (PCFI), Comparative Fit Index (CFI), Parsimonious Normed Fit Index (PNFI), and root-mean-squared error of approximation (RMSEA). Acceptable model fit is indicated by *χ*^2^/df < 5.0 (<3.0 good), CFI, IFI, GFI > 0.9, PNFI, PCFI > 0.5, and RMSEA < 0.08 [41]. Two thousand bootstrap resamples were used to determine the role of mindfulness as a moderator variable between perceived stress and quality of life. The significance level was set at 0.05.

### 2.6. Ethical Considerations

The code of ethics (IR.KMU.REC.1398.390) was received from the Ethics Committee of Kerman University of Medical Sciences. In this project, the purpose of the research was fully explained to the participants who could withdraw from the study at any time. They were explained that participating in or withdrawing from the study would not affect their treatment process in any way, and all their information would remain confidential.

## 3. Results

The mean age of the study participants was 50.49 ± 15.27 years. The majority of the samples were male, married, educated, and employed. The monthly income level of most samples was <2 million tomans a month (13000 tomans = one dollar). Eighteen percent of the patients had been diagnosed with cancer for more than two years. The majority of the participants had breast or respiratory or blood cancers. The majority of the participants had no other history of chronic disease (Table 1).

The mean score of mindfulness was 68.41 ± 13.12, which was greater than the midpoint of the questionnaire (52.5). The mean score of perceived stress was 27.61 ± 7.23, which was greater than the midpoint of the questionnaire (28). The mean scores of overall quality of life, functioning, and symptom were 56.34, 61.24, and 38.71, respectively (Table 2).

No significant correlation was found between mindfulness and age (*r* = 0.005, *P* = 0.95). Mindfulness was significantly greater in patients who had been diagnosed with cancer for less than two years than those above two years (*P*=0.005). The Bonferroni post hoc test showed that mindfulness was not different between patients who had been diagnosed with cancer for one or two years (*P*=0.81), but it was significantly lower in patients who had been diagnosed with cancer for above two years than those with one year (*P*=0.008) or two years (*P*=0.01). Mindfulness score was not different among other qualitative variables (Table 1).

Mindfulness was negatively correlated with perceived stress, subscale of PSS-NW, and symptom subscale of QOL. Mindfulness was positively correlated with the functioning subscale of QOL. No significant correlation was found between mindfulness and overall quality of life (Table 2).

Table 3 shows that the proposed model has relatively good fit indices. The model was modified by drawing the correlation between the measurement errors e5 and e7 (Table 3).

The results showed that the *R*^2^ for quality of life was 0.384, indicating that all independent and mediating variables, perceived stress and mindfulness, can predict nearly 39% of the variances in QOL of the cancer patients.

**Figure 1:**
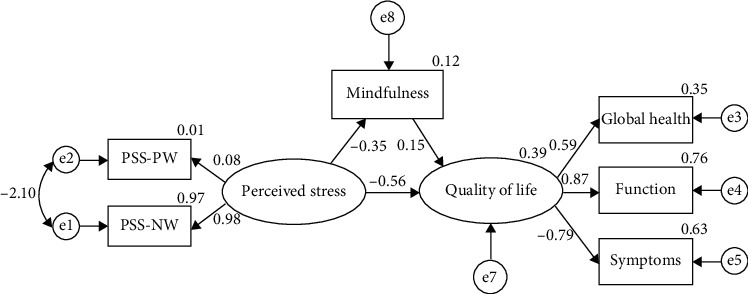
Standard coefficients of the modified model.

The results of the path analysis showed a significantly direct correlation between the variables. Perceived stress was negatively associated with mindfulness and quality of life (*P* < 0.05). In addition, mindfulness was positively associated with quality of life (*P* < 0.05) (Table 4) (Figure 1). Mindfulness played a mediating role in the relationship between perceived stress and quality of life (Table 4).

## 4. Discussion

The current study found a positive correlation between mindfulness and functioning (a dimension of QOL) and a negative correlation between mindfulness, symptoms (a dimension of QOL), and perceived stress.

The results of some studies have also shown that mindfulness can increase the quality of life of cancer patients [6, 30, 31]. Past studies have shown that mindfulness training is closely correlated with the development of attentional functions [44], cognitive flexibility [45], and problem solving [46], and such factors can affect and improve patients' condition. In addition, Poulin et al. found that mindfulness improved the two aspects of nonjudgment and acting with awareness of the cancer patients, which prevented patients' fear and helped them get better results in life. Poulin et al. showed that mindfulness reduced pain of cancer patients and made them better adapt to the disease and increased their quality of life [31]. All of these results suggest that mindfulness can help cancer patients overcome their fear and better adapt to, affect, and improve different aspects of life, including the quality of life. However, Lengacher et al. showed that mindfulness did not improve quality of life of these patients [32]. These results could be due to the study of various variables in this study, and the simultaneous study of patients and caregivers can also affect the results.

The present study showed that the higher the mindfulness of the cancer patients, the lower their perceived stress. Hsieh et al. indicated that mindfulness of the cancer patients was negatively correlated with their stress [47]. This result has been confirmed in several studies [29, 32, 48, 49]. These results highlight the importance of mindfulness in reducing mood disorders and stress of the cancer patients. Cancer patients who are more mindful can be more aware of their experiences and more self-accepting [50], which can help them satisfy with their values and beliefs [51] and reduce their anxiety, confusion, and stress. Bao et al. showed that mindfulness activated an important behavioral mechanism called regulation of emotion (ROE), which might reduce perceived stress. Mindfulness is also a mediator between use of emotion (UOE) and perceived stress [48], meaning that people with a high level of mindfulness use their emotions more to improve functioning, which help reduce perceived stress. Lengacher et al. found that mindfulness affected certain stress hormones (e.g., cortisol) and cytokine levels (e.g., IL-6), stress, and immune system function [32]. These results suggest that people with high levels of mindfulness have more control over stress because of the mediators affecting the body emotions and physiological system.

As we proposed, the results showed that mindfulness could buffer the impact of perceived stress on QOL. In other words, the quality of life of the cancer patients with perceived stress will increase as they become more mindful. Results of the current study may reveal the process of how mindfulness can improve QOL of the patients with cancer.

Hsieh et al. also showed that mindfulness in people who were aware of the factors affecting stress could moderate the perceived stress [47]. Despite the effectiveness of mindfulness in various psychological disorders, it is unclear how mindfulness regulates the emotion and stress. Shapiro et al. believe that mindfulness may act with changes in attention, intention, and attitude [52]. Others argue that the positive effects of mindfulness can be explained by mechanisms such as observing, describing, acting with awareness, not judging internal experiences, and not responding to internal experiences [53]. These results suggest that people who are more mindful can focus on current problems and remove negative thoughts that reduce perceived physical stress and mental stress and lead to improved quality of life.

Hölzel et al. suggested that mindfulness could regulate attention, body awareness, emotion, and self-perspectives through gentle changes in brain function and thus could influence on individuals' health [54]. The main goal of mindfulness programs is to correct dysfunctional individual strategies and regulate emotions, so it may help people regulate their emotional capacities that lead to symptomatic and clinical recovery and improve people's quality of life. Furthermore, Poulin et al. showed that mindfulness reduced pain intensity, pain catastrophizing, pain interference, and depression of cancer patients and increased their quality of life [31]. The results of the present study emphasize that mindfulness can improve the quality of life of cancer patients by reducing perceived stress. Therefore, strengthening mindfulness should be considered as one of the effective mechanisms in controlling the stress of cancer patients. It is also necessary to pay attention and understand the factors affecting mindfulness to improve the quality of life of the cancer patients.

The present study showed no correlation between mindfulness and age, which is consistent with previous studies [6, 30, 47]. In addition, patients who had been diagnosed with cancer for less than two years were more mindful compared with other cancer patients. One reason can be patients' disappointment and fatigue because of the disease recurrence, so they did not consider medical issues. Al-Ghabeesh et al. found that the quality of life of cancer patients reduced over time [6]. The reason can be long-term involvement of the patients with metastatic disease or their exposure to a growing number of diseases that affect their psychological functioning and keep patients away from mindfulness issues. The results of the studies suggest that patients who are more mindful may be better able to regulate their emotional responses [55], which can help the patients cope with the disease and improve their living conditions and quality of life.

The results of this study show that a high level of mindfulness is important in reducing the cancer patients' stress and some aspects of quality of life. Given the direct mediating role of mindfulness, this study recommends the use of mindfulness training in reducing some of the consequences of cancer, such as stress. Physicians can use mindfulness-based interventions in supportive therapies in addition to the main treatments of the disease, with the aim of empowering patients and improving their acute condition, especially reducing their psychological symptoms. Therefore, a mindfulness-based program aiming at improving some aspects of quality of life and reducing stress can strengthen cancer patients for long-term problems and have a significant impact on their lives. In addition, nurses and physicians, the main caregivers of patients, should pay attention to the mindfulness program that can be effective in implementing mindfulness interventions by patients and their empowerment.

There are limitations in our study that can be addressed in future research. Since the present study is cross-sectional and does not examine the cause-and-effect relationship, the future research should be conducted in the form of longer-term longitudinal designs or an intervention study. Results should be generalized with caution because the study population was the cancer patients in two governmental centers and their specific condition and long course of treatment can affect the mindfulness, perceived stress, and quality of life of these patients.

## 5. Conclusion

The results of this study showed that the variables of mindfulness and perceived stress affected the quality of life of cancer patients. Mindfulness can directly affect the quality of life of cancer patients, indirectly reduce their stress, and potentially affect their quality of life. According to the results, it is suggested that special attention be paid to mindfulness constructs to increase the quality of life, the power to deal effectively with mental stress and tensions, and empower the cancer patients. It is also suggested that the present study be conducted on other sample groups, including normal and healthy individuals and other age groups, and that future research should focus on the dimensions and components of mindfulness. The correlation between other psychological factors and the components of mindfulness should also be examined.

## Figures and Tables

**Table 1:** Demographic and clinical characteristics of the participants and mindfulness differences among the participants.

	Variable	Frequency (%)	Mindfulness		Statistic test	*P* value
			Mean	SD		
Sex	Female	93 (45.4)	67.60	13.86	*t* = 0.80	0.42
	Male	112 (54.6)	69.08	12.49		

Marital status	Single	25 (12.2)	70.52	11.09	*F* = 1.66	0.19
	Married	168 (82.0)	68.54	13.36		
	Divorced/widow(er)	12 (5.9)	62.25	12.71		

Education level	Uneducated	64 (31.2)	66.48	15.0	*H* = 1.13	0.77
	Middle/high school	56 (27.3)	69.41	11.58		
	Diploma	33 (16.1)	67.45	15.46		
	Academic	52 (25.4)	70.31	10.28		

Job	Employed	120 (58.5)	68.99	12.0	*Z* = −0.18	0.86
	Unemployed	85 (41.5)	67.59	14.58		

Income (million tomans)	<1	122 (59.5)	67.78	14.08	*F* = 0.74	0.53
	1–2	32 (15.6)	67.59	12.17		
	2–3	32 (15.6)	69.31	12.42		
	>3	19 (9.3)	72.32	8.74		

History of cancer (month)	≤12	136 (66.4)	69.47	12.82
	13–24	32 (15.6)	71.03	12.34	*F* = 5.39	0.005
	>24	37 (18.0)	62.24	13.35		

Type of cancer	Breast	40 (19.5)	67.05	13.37	*F* = 0.29	0.94
	Gastrointestinal	23 (11.2)	66.78	11.45		
	Respiratory	34 (16.6)	68.03	15.14		
	Blood	33 (16.1)	69.42	15.06		
	Lymphoma	21 (10.2)	70.76	12.037		
	Bone marrow	22 (10.7)	69.14	12.66		
	Other	32 (15.6)	68.59	10.94		

Cancer stage	Ι	92 (44.9)	70.01	13.12	*F* = 1.30	0.28
	Π	71 (34.6)	66.80	11.71		
	ΠΙ	42 (20.5)	67.62	15.13		

Cancer treatment	Chemotherapy	100 (48.8)	68.98	12.88	*F* = 0.22	0.80
	Surgery and chemotherapy	57 (27.8)	68.18	12.75		
	Other combined therapies	48 (23.4)	67.50	14.23		

History of chronic disease	Yes	70 (34.1)	69.25	12.92	*t* = 1.28	0.20
	No	135 (65.9)	66.79	13.44		

*t* = independent *t*-test; *F* = ANOVA; *H* = Kruskal–Wallis H; *Z* = Mann–Whitney U.

**Table 2:** The correlation among mindfulness, perceived stress, and quality of life of the patients with cancer.

Variable	Mean (SD)	Pearson correlation coefficient						
		1	2	3	4	5	6	7
1. Mindfulness	68.41 (13.12)	1						
2. Perceived stress	27.61 (7.23)	−0.27 (*P* < 0.001)	1					
3. Perceived stress (positive word)	12.31 (6.31)	−0.03 (*P*=0.68)	0.65 (*P* < 0.001)	1				
4. Perceived stress (negative word)	15.30 (5.73)	−0.31 (*P* < 0.001)	0.55 (*P* < 0.001)	−0.28 (*P* < 0.001)	1			
5. Global quality of life	56.34 (24.94)	0.12 (*P*=0.09)	−0.41 (*P* < 0.001)	−0.20 (*P*=0.004)	−0.30 (*P* < 0.001)	1		
6. Functioning (QOL)	61.24 (20.08)	0.36 (*P* < 0.001)	−0.49 (*P* < 0.001)	−0.10 (*P*=0.14)	−0.50 (*P* < 0.001)	0.50 (*P* < 0.001)	1	
7. Symptom (QOL)	38.71 (20.99)	−0.22 (*P*=0.002)	0.33 (*P* < 0.001)	−0.06 (*P*=0.36)	0.49 (*P* < 0.001)	−0.50 (*P* < 0.001)	−0.69 (*P* < 0.001)	1

SD: standard deviation; QOL: quality of life.

**Table 3:** Fit indices of the primary and modified model.

Model indices	*χ* ^2^	df	*χ* ^2^/df	*P* value	GFI	IFI	PCFI	CFI	PNFI	RMSEA
Primary model	39.03	8	4.88	<0.001	0.94	0.90	0.48	0.90	0.47	0.08
Modified model	20.87	7	2.98	<0.001	0.96	0.94	0.57	0.94	0.55	0.07

*χ* ^2^: chi-square; df: degree of freedom; GFI: Goodness of Fit Index; IFI: Incremental Fit Index; PCFI: Parsimonious Comparative Fit Index; CFI: Comparative Fit Index; PNFI: Parsimonious Normed Fit Index; RMSEA: root–mean-square error of approximation.

**Table 4:** Path analysis on the direct and indirect effects of the mindfulness on perceived stress and quality of life among patients with cancer.

Direct Path	Standardized *β*	SE	CR	*P* value
Mindfulness <-- perceived stress	−0.35	0.13	−5.42	<0.001
Quality of life <-- mindfulness	0.15	0.08	2.21	0.03
Quality of life <-- perceived stress	−0.56	0.22	−6.28	<0.001

Indirect path	Standardized *β*	SE	CI	*P* value
Quality of life <-- perceived stress <-- mindfulness	−0.13	0.07	−0.28 to −0.01	0.04

SE: standard error; CR: critical ratio; CI: confidence interval.

## Data Availability

Data are available from the corresponding author upon request.
